# Cruciform structures are a common DNA feature important for regulating biological processes

**DOI:** 10.1186/1471-2199-12-33

**Published:** 2011-08-05

**Authors:** Václav Brázda, Rob C Laister, Eva B Jagelská, Cheryl Arrowsmith

**Affiliations:** 1 Institute of Biophysics, Academy of Sciences of the Czech Republic, v.v.i., Královopolská 135, Brno, 612 65, Czech Republic; 2 Division of Stem Cell and Developmental Biology, Department of Medical Biophysics, Ontario Cancer Institute, University of Toronto, 101 College Street, Toronto, Ontario, M5G 1L7, Canada; 3 Cancer Genomics & Proteomics, Department of Medical Biophysics, Ontario Cancer Institute, University of Toronto, 101 College Street, Toronto, Ontario, M5G 1L7, Canada

**Keywords:** cruciform structure, inverted repeat, protein-DNA binding

## Abstract

DNA cruciforms play an important role in the regulation of natural processes involving DNA. These structures are formed by inverted repeats, and their stability is enhanced by DNA supercoiling. Cruciform structures are fundamentally important for a wide range of biological processes, including replication, regulation of gene expression, nucleosome structure and recombination. They also have been implicated in the evolution and development of diseases including cancer, Werner's syndrome and others.

Cruciform structures are targets for many architectural and regulatory proteins, such as histones H1 and H5, topoisomerase IIβ, HMG proteins, HU, p53, the proto-oncogene protein DEK and others. A number of DNA-binding proteins, such as the HMGB-box family members, Rad54, BRCA1 protein, as well as PARP-1 polymerase, possess weak sequence specific DNA binding yet bind preferentially to cruciform structures. Some of these proteins are, in fact, capable of inducing the formation of cruciform structures upon DNA binding. In this article, we review the protein families that are involved in interacting with and regulating cruciform structures, including (a) the junction-resolving enzymes, (b) DNA repair proteins and transcription factors, (c) proteins involved in replication and (d) chromatin-associated proteins. The prevalence of cruciform structures and their roles in protein interactions, epigenetic regulation and the maintenance of cell homeostasis are also discussed.

## Review

Genome sequencing projects have inundated us with information regarding the genetic basis of life. While this wealth of information provides a foundation for our understanding of biology, it has become clear that the DNA code alone does not hold all the answers. Epigenetic modifications and higher order DNA structures beyond the double helix also contribute to basic biological processes and maintaining cellular stability. Local alternative DNA structures are known to exist in all life forms [1]. The negative supercoiling of DNA can induce local nucleotide sequence-dependent conformational changes that give rise to cruciforms, left-handed DNA, triplexes and quadruplexes [2-4]. The formation of cruciforms is strongly dependent on base sequence and requires perfect or imperfect inverted repeats of 6 or more nucleotides in the DNA sequence [5,6]. Over-representation of inverted repeats, which occurs nonrandomly in the DNA of all organisms, has been noted in the vicinity of breakpoint junctions, promoter regions, and at sites of replication initiation [3,7,8]. Cruciform structures may affect the degree of DNA supercoiling, the positioning of nucleosomes *in vivo *[9], and the formation of other secondary structures of DNA. Cruciforms contain a number of structural elements that serve as direct protein-DNA targets. Numerous proteins have been shown to interact with cruciforms, recognizing features such as DNA crossovers, four-way junctions, and curved or bent DNA. Structural transitions in chromatin occur concomitantly with DNA replication or transcription and in processes that involve a local separation of DNA strands. Such transitions are believed to facilitate the formation of alternative DNA structures [10,11]. Transient supercoils are formed in the eukaryotic genome during DNA replication and transcription, and these often involve protein binding [12]. Indeed, active chromatin remodeling is a typical feature for many promoters and is essential for gene transcription [13]. Notably, DNA supercoiling can have a strong impact on gene expression [14]. Using microarrays covering the *E. coli *genome, it was recently shown that expression of 7% of genes was rapidly and significantly affected by a loss of chromosomal supercoiling [15]. Several complexes that involve extensive DNA-protein interactions, whereby the DNA wraps around the protein, can only occur under conditions of negative DNA supercoiling [10]. Other proteins are reported to interact with the supercoiled DNA (scDNA) at crossing points or on longer segments of the interwound supercoil [16,17]. Interestingly, the eukaryotic genome has been shown to contain a percentage of unconstrained supercoils, part of which can be attributed to transcriptional regulation [3]. The spontaneous generation of DNA supercoiling is also a requirement for genome organization [18]. Transient supercoils are formed both in front of and behind replication forks as superhelical stress is distributed throughout the entire replicating DNA molecule [19]. A number of additional processes may operate to create transient and localized superhelical stresses in eukaryotic DNA.

The recognition of cruciform DNA seems to be critical not only for the stability of the genome, but also for numerous, basic biological processes. As such, it is not surprising that many proteins have been shown to exhibit cruciform structure-specific binding properties. In this review, we focus on these proteins, many of which are involved in chromatin organization, transcription, replication, DNA repair, and other processes. To organize our review, we have divided cruciform binding proteins into four groups (see Table 1) according to their primary functions: (a) junction-resolving enzymes, (b) transcription factors and DNA repair proteins, (c) replication machinery, and (d) chromatin-associated proteins. For each group, we describe in detail recent examples of research findings. Lastly, we review how dysregulation of cruciform binding proteins is associated with the pathology of certain diseases found in humans.

**Table 1:** Proteins involved in interactions with cruciform structures

*Protein*	*Source*	*Reference*
**Junction-resolving enzymes**		
Integrase family		
RuvC	E.coli	[133-135]
Cce1	yeast	[136]
Ydc2	S.pombe	[134]
A22	Coccinia virus	[137]
Integrases	all	[119,138]
Restriction nuclease family		
Endonuclease I	Phage T7	[139-141]
RecU	G+ bacteria	[134,142]
Hjc, Hje	archea	[134,143]
MutH	Eukaryotes	[25,144]
Other		
Endonuclease VII	phage T4	[25,145]
RusA	E.coli	[146]
MSH2	S. cerevisiae	[147,148]
Mus81-Eme1	Eukaryotes	[42,149-151]
TRF2	H. sapiens	[52,152]
XPF, XPG protein families	Eukaryotes	[56,153,154]

**Transcription, Transcription factors and DNA repair**		
PARP-1	H. sapiens and others	[51,63]
BRCA1	H. sapiens and others	[49,50,91,93]
P53	H. sapiens and others	[69,73,75,76,132,155,156]
Bmh1	S.cerevisiae	[35]
14-3-3	H. sapiens, S.cerevisiae	[34,110]
Rmi-1	Yeast	[157]
Crp-1	S. cerevisiae	[158]
HMG protein family	all	[47,159-161]
Smc	S. cerevisiae	[118,162]
Hop1	S. cerevisiae	[163,164]
ER estrogen receptor	mammals	[58]

**Chromatin-associated proteins**		
DEK	mammals	[84,85]
BRCA1	mammals	[49,50,91,93]
HMG protein family	Eukaryotes	[47,159-161]
Rad54	Eukaryotes	[48]
Rad51ap	Eukaryotes	[81]
Topoisomerase I	Eukaryotes	[101,165]

**Replication**		
S16	E.coli	[113]
GF14, homolog of 14-3-3	plants	[35]
MLL (leukemia)	H. sapiens	[125,126]
WRN (Werner syndrome)	H. sapiens	[129]
AF10	H. sapiens	[114]
14-3-3	Eukaryotes	[34,110]
DEK	mammals	[84,85]
DNA-PK	Eukaryotes	[166]
Vlf-1	Baculovirises	[119]
HU	E. coli	[105,167,168]
Helicases (59, 44, and others)	all	[55]

### Formation and presence of *c*ruciform structures in the genome

**Figure 1:**
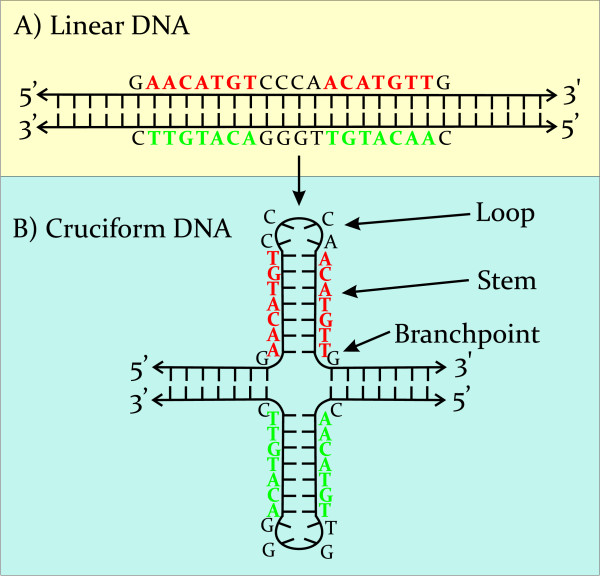
**Changes associated with transition from the linear to cruciform state in the p53 target sequence from the p21 promoter**. The promoter sequence contains a 20 bp p53 target sequence with 7 bp long inverted repeat (red), (A) as linear DNA and (B) as an inverted repeat as a cruciform structure. In the cruciform structure, the p53 target sequence is presented as stems and loops.

**Figure 2:**
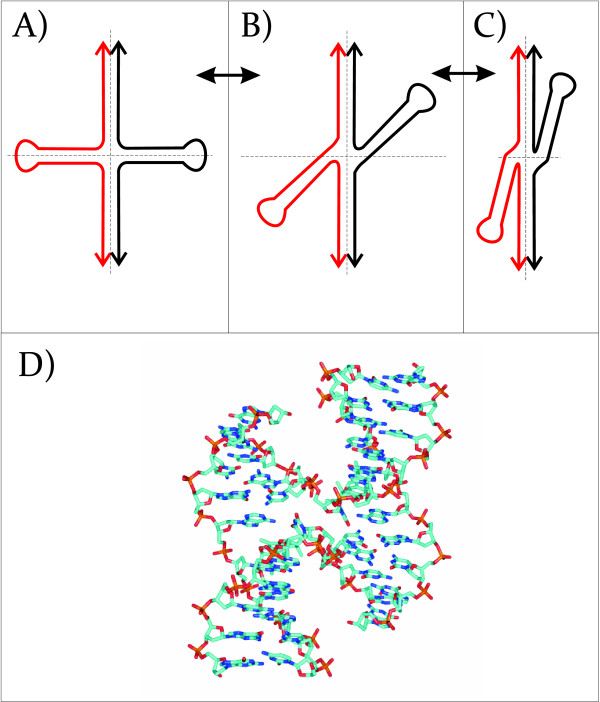
**Conformations of a cruciform structure**. Conformations of a cruciform can vary from (A) "unfolded" with 4-fold symmetry to (B) bent, and to (C) "stacked" with 4 chains of DNA in close vicinity. D) Topology of a Holliday junction stabilized by a psoralen cross-linking agent (PDBID 467D). Here, the junction takes the form of an anti-parallel stacked x-structure.

Cruciform structures are important regulators of biological processes [3,5]. Both stem-loops and cruciforms are capable of forming from inverted repeats. Cruciform structures consist of a branch point, a stem and a loop, where the size of the loop is dependent on the length of the gap between inverted repeats (Figure 1). Direct inverted repeats lead to formation of a cruciform with a minimal single-stranded loop. The formation of cruciforms from indirect inverted repeats containing gaps is dependent not only on the length of the gap, but also on the sequence in the gap. In general, the AT-rich gap sequences increase the probability of cruciform formation. It is also possible that the gap sequence can form an alternative DNA structure. The formation of DNA cruciforms has a strong influence on DNA geometry whereupon sequences that are normally distal from one another can be brought into close proximity [20,21]. The structure of cruciforms has been studied by atomic force microscopy [22-24]. These studies have identified two distinct classes of cruciforms. One class of cruciforms, denoted as unfolded, have a square planar conformation characterized by a 4-fold symmetry in which adjacent arms are nearly perpendicular to one another. The second class comprises a folded (or stacked) conformation where the adjacent arms form an acute angle with the main DNA strands (Figure 2). Two of the three structural motifs inherent to cruciforms, the branch point and stem, are also found in Holliday junctions. Holliday junctions are formed during recombination, double-strand break repair, and fork reversal during replication. Resolving Holliday junctions is a critical process for maintaining genomic stability [25,26]. These junctions are resolved by a class of structure-specific nucleases: the junction-resolving enzymes.

Cruciforms are not thermodynamically stable in naked linear DNA due to branch migration [27]. Cruciform structure formation *in vivo *has been shown in both prokaryotes and eukaryotes using several methodological approaches. The presence of the cruciform structure was first described in circular plasmid DNA where the negative superhelix density can stabilize cruciform formation. Plasmids with native superhelical density usually contain cruciform structures *in vitro *and *in vivo *[28]. For example, higher order structure in the pT181 plasmid was shown to exist *in vivo *using bromoacetaldehyde treatment [29]. Deletion of the sequence which forms this structure at the ori site leads either to a reduction or failure in replication [30]. Similarly, deletion of the cruciform binding domain in 14-3-3 proteins results in reduced origin binding which affects the initiation of DNA replication in budding yeast [31]. Monoclonal antibodies against cruciform structures have also been used successfully to isolate cruciform-containing segments of genomic DNA. Furthermore, these sequences were able to replicate autonomously when transfected into HeLa cells [32]. Stabilization of the cruciform structures by monoclonal antibodies 2D3 and 4B4, with anti-cruciform DNA specificity, resulted in a 2- to 6-fold enhancement of replication *in vivo *[33]. 14-3-3 sigma was found to associate *in vivo *with the monkey origins of DNA replication ors8 and ors12 in a cell cycle-dependent manner, as assayed by a chromatin immunoprecipitation (ChIP) assay that involved formaldehyde cross-linking, followed by immunoprecipitation with anti-14-3-3 sigma antibody and quantitative PCR [34]. Similarly, the 14-3-3 protein homologs from *Saccharomyces cerevisiae*, Bmh1p and Bmh2p, have cruciform DNA-binding activity and associate *in vivo *with ARS307 [35]. Several studies show that transcription is regulated directly by the presence of cruciform structure *in vivo*. Another example includes the ability of the d(AT)n-d(AT)n insert to spontaneously adopt a cruciform state in *E. coli*, resulting in a block of protein synthesis [36]. Using site-directed mutational analysis and P1 nuclease mapping, it was demonstrated that the formation of a cruciform structure is required for the repression of enhancer function in transient transfection assays and that Alu elements may contribute to regulation of the CD8 alpha gene enhancer through the formation of secondary structure that disrupts enhancer function [37]. Transcriptionally driven negative supercoiling also mediates cruciform formation *in vivo *and enhanced cruciform formation correlates with an elevation in promoter activity [38]. It was also shown that the secondary DNA structures of the ATF/CREB element play a vital role in protein-DNA interactions and its cognate transcription factors play a predominant role in the promoter activity of the RNMTL1 gene [39]. Hypo-methylation of inverted repeats by the Dam methylase show that these sequences are consistent with an unusual secondary structure, such as DNA cruciform or hairpin *in vivo *[40]. The *in vivo *effects of cruciform formation during transcription have been studied in detail by Krasilnikov et al. [4]. Interestingly hairpin-capped linear DNA (in which the replication of hairpin-capped DNA and cruciform formation and resolution play central roles) was stably maintained for months in a human cancer cell line as numerous extra-chromosomal episomes [41]. Long palindromes can also induce DNA breaks after assuming a cruciform structure. Palindromes in *S. cerevisiae *are resolved, *in vivo*, by structure-specific enzymes. In vivo resolution requires either the Mus81 endonuclease or, as a substitute, the bacterial HJ resolvase RusA. These findings provide confirmation of cruciform extrusion and resolution in the context of eukaryotic chromatin [42]. Taken together, these studies show that cruciforms have been detected *in vivo *using a variety of independent techniques and that they are an intriguing and integral phenomenon of DNA biology and biochemistry.

### Proteins involved in interactions with cruciform structures

#### Junction-resolving enzymes

**Figure 3:**
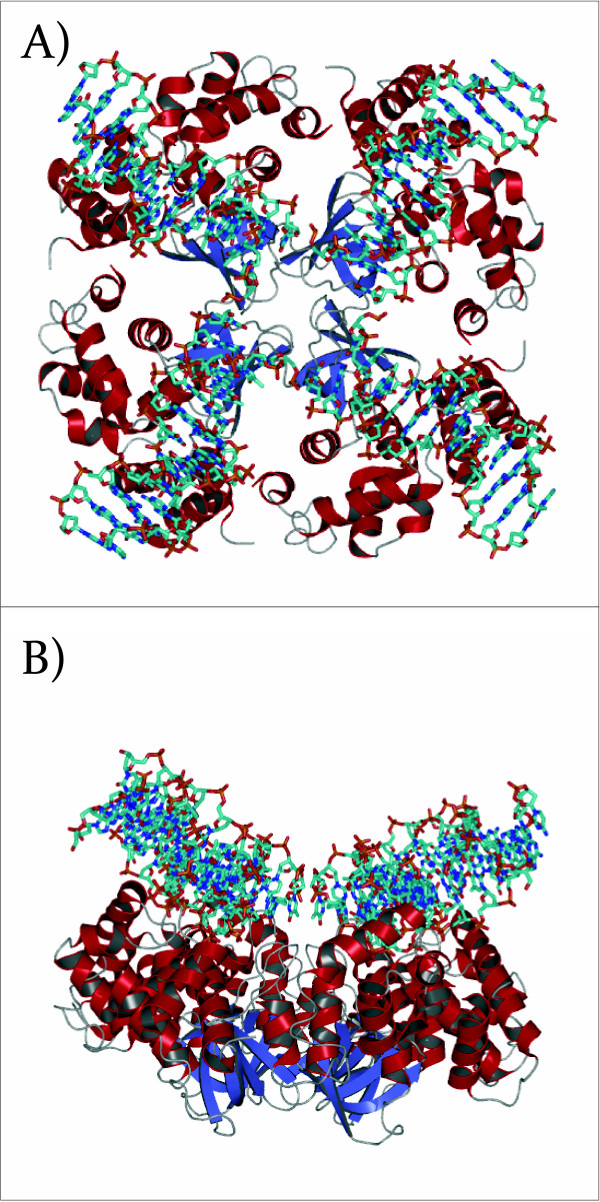
**Crystal structure of the E. coli RuvA tetramer in complex with a Holliday junction (PDBID **1C7Y**)**. A) The Holliday junction is depressed at the center where it makes close contacts with RuvA. Each of the arms outside of the junction center takes on a standard beta-DNA conformation B) Rotation of A) by 90°.

There are a large number of proteins that recognize cruciforms (summarized in Table 1) and, of these, the junction-resolving enzymes have been studied extensively. These proteins have been identified in many organisms from bacteria (and their phages) to yeast, archea and mammals [43]. The majority of the junction-resolving enzymes can be divided into one of two superfamilies [44]. Those in the first class target specific DNA sequences for enzymatic activity, although they will bind equally well to junctions of any sequence. This superfamily includes E. coli RuvC, the yeast integrases, Cce1, Ydc2, and RnaseH. The second group includes the phage T7, endonuclease RecU, the Hjc and Hje resolving enzymes, the MutH protein family and related restriction enzymes. The x-ray structures of the junction-resolving enzymes in complex with 4-way junctions highlight the flexibility inherent to DNA (Figure 3) [25] in that these enzymes recognize and distort the junction. This enables them to carry out such key roles as the cleavage of allogene DNAs and maintenance of genomic stability to name but a few. The recognition of non-B-DNA structure by junction-resolving enzymes has been the subject of several reviews [25,43,45,46].

#### Proteins involved in transcription and DNA repair

The maintenance of a cell's genomic stability is achieved through several independent mechanisms. Arguably, the most important of these mechanisms is DNA repair. Protein binding to damaged DNA and to the local alternative DNA structures is therefore a key function of these processes. The promoter regions of genes are often characterized by presence of inverted repeats that are capable of forming cruciforms *in vivo*. A number of DNA-binding proteins, such as those of the HMGB-box family [47], Rad54 [48], BRCA1 protein [49,50], as well as PARP-1 (poly(ADP-ribose) polymerase-1) [51], display only a weak sequence preference but bind preferentially to cruciform structures. Moreover, some proteins can induce the formation of cruciform structures upon DNA binding [51,52]. Among the DNA repair proteins which bind to cruciforms are the junction-resolving enzymes Ruv and RuvB [53,54], DNA helicases [55], XPG protein [56], and multifunctional proteins like HMG-box proteins [57] BRCA1, 14-3-3 protein family including homolog's Bmh1 and Bmh2 from *S. cerevisiae*, and GF14 from plants. Footprinting analysis of the gonadotropin-releasing hormone gene promoter region indicated the human estrogen receptor (ER) to be another potential cruciform binding protein. In this case, extrusion of the cruciform structure allowed the estrogen response elements motifs to be accessed by the ER protein [58].

##### PARP-1

**Figure 4:**
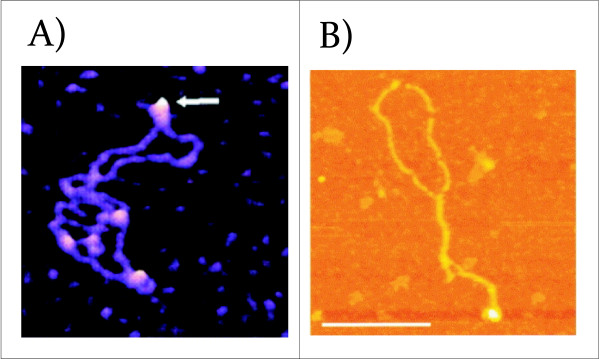
**AFM and SFM images of proteins binding to a cruciform structure**. A) AFM images of PARP-1 binding to supercoiled pUC8F14 plasmid DNA containing a 106 bp inverted repeat. PARP-1 binds to the end of the hairpin arm (white arrow). Images show 300 × 300 nm^2 ^surface areas (reprinted with permission from [51]. B) The interaction between p53CD and supercoiled DNA gives rise to cruciform structures. Shown is an SFM image of complex formed between p53CD and sc pXG(AT)_34 _plasmid DNA at a molar ratio of 2.5; the complexes were mounted in the presence of 10 mM MgAc_2_. The scale bars represent 200 nm (reprinted with permission from [132].

PARP-1 is an abundant, nuclear, zinc-finger protein present in ~ 1 enzyme per 50 nucleosomes. It has a high affinity for damaged DNA and becomes catalytically active upon binding to DNA breaks [59]. In the absence of DNA damage, the presence of PARP-1 leads to the perturbation of histone-DNA contacts allowing DNA to be accessible to regulatory factors [60]. PARP-1 activity is also linked to the coordination of chromatin structure and gene expression in Drosophila [61]. It was reported that PARP can bind to the DNA hairpins in heteroduplex DNA and that the auto-modification of PARP in the presence of NAD+ inhibited its hairpin binding activity. Atomic force microscopy studies revealed that, *in vitro*, PARP protein has a preference for the promoter region of the PARP gene in superhelical DNA where the dyad symmetry elements form hairpins (Figure 4) [62]. PARP-1 recognizes distortions in the DNA backbone allowing it to bind to three- and four-way junctions [63]. Kinetic analysis has revealed that the structural features of non-B form DNA are important for PARP-1 catalysis activated by undamaged DNA. The order of PARP-1's substrate preference has been shown to be: cruciforms > loops > linear DNA. These results suggest a link between PARP-1 binding to cruciforms structures in the genome and its function in the modulation of chromatin structure in cellular processes. Moreover, it was shown that the binding of PARP-1 to DNA can induce changes in DNA topology as was demonstrated using plasmid DNA targets [51].

##### P53

P53 is arguably one of the most intensively studied tumor suppressor genes. More than 50% of all human tumors contain p53 mutations and the inactivation of this gene plays a critical role in the induction of malignant transformation [64]. Sequence-specific DNA binding is crucial for p53 function. P53 target sequences, which consist of two copies of the sequence 5'-RRRC(A/T)(T/A)GYYY-3, often form inverted repeats [65]. It was reported that p53 binding is temperature sensitive and dependent on DNA fragment length [66,67]. Moreover, it was demonstrated, *in vivo*, that p53 binding to its target sequence is highly dependent on the presence of an inverted repeat at the target site. Preferential binding of p53 to superhelical DNA has also been described [68,69]. Non-canonical DNA structures such as mismatched duplexes, cruciform structures [70], bent DNA [71], structurally flexible chromatin DNA [13], hemicatenated DNA [72], DNA bulges, three- and four-way junctions [73], or telomeric t-loops [74] can all be bound selectively by p53. There is a strong correlation between the cruciform-forming targets and an enhancement of p53 DNA binding [75]. Target sequences capable of forming cruciform structures in topologically constrained DNA bound p53 with a remarkably higher affinity than did the internally asymmetrical target site [76]. These results implicate DNA topology as having an important role in the complex, with possible implications in modulation of the p53 regulon.

#### Chromatin-associated proteins

The chromatin-associated proteins cover a broad spectrum of the proteins localized in the cell nucleus. They are partly involved in modulating chromatin structure, but are also implicated in a range of processes associated with DNA function. They fine-tune transcriptional events (DEK, BRCA1) and are involved in both DNA repair and replication (HMG proteins, Rad51, Rad51ap, topoisomerases). Another family of enzymes deemed important in these processes is that of topoisomerases. These enzymes occur in all known organisms and play crucial roles in the remodeling of DNA topology. Topoisomerase I binds to Holliday junctions [77], and topoisomerase II recognizes and cleaves cruciform structures [78] and interacts with the HMGB1 protein [57]. These processes are particularly important for maintaining genomic stability due to their ability to diffuse the stresses that are levied upon a DNA molecule during transcription, replication and the resolving of long cruciforms that would otherwise hinder DNA chain separation. The Rad54 protein plays an important role during homologous recombination in eukaryotes [79]. Yeast and human Rad54 bind specifically to Holliday junctions and promote branch migration [80]. The binding preference for the open conformation of the X-junction appears to be common for many proteins that bind to Holliday junctions. Human Rad54 binds preferentially to the open conformation of branched DNA as opposed to the stacked conformation [48]. Similarly, RAD51AP1, the RAD51 accessory protein, specifically stimulates joint molecule formation through the combination of structure-specific DNA binding and by interacting with RAD51. RAD51AP1 has a particular affinity for branched-DNA structures that are obligatory intermediates during joint molecule formation [81]. The recognition of branched structures during homologous recombination is a critical step in this process.

##### DEK

The human DEK protein is an abundant nuclear protein of 375 amino acids that occurs in numbers greater than 1 million copies per nucleus [82]. Its interactions with transcriptional activators and repressors suggest that DEK may have a role in the formation of transcription complexes at promoter and enhancer sites [reviewed in [83]]. The binding of DEK to DNA is not sequence specific and DEK has a clear preference for supercoiled and four-way junctions [84]. Work with isolated and recombinant DEK has shown that it has intrinsic DNA-binding activity with a preference for four-way junction and superhelical DNA over linear DNA and introduces positive supercoils into relaxed circular DNA [83,85]. DEK has two DNA-binding domains. The first domain is centrally located and harbors a conserved sequence element, the SAF (scaffold attachment factor). The second DNA-binding domain is located at the C-terminus of DEK which is also post-translationally modified by phosphorylation. In fact, the DNA-binding properties of DEK are clearly influenced by phosphorylation as phosphorylated DEK binds with a weaker affinity to DNA than does unmodified DEK and induces the formation of DEK multimers [86,87]. DEK's monomeric SAF box (residues 137-187) does not appear to interact with DNA in solution. However, when many SAF boxes are brought into close proximity, it cooperativity drives DNA binding. A DEK construct spanning amino acids 87-187 binds to DNA much like the intact DEK preferring four-way DNA junctions over linear DNA. This fragment forms large aggregates in the presence of DNA and is also able to introduce supercoils into relaxed circular DNA. Interestingly, the 87-187 amino acid peptide induces negative DNA supercoils [88].

##### BRCA1

BRCA1 is a multifunctional tumor suppressor protein having roles in cell cycle progression, transcription, DNA repair and chromatin remodeling. Mutations to the BRCA1 gene are associated with a significant increase in the risk of breast cancer. The function of BRCA1 likely involves interactions with both DNA and an array of proteins. BRCA1 associates directly with RAD51 and both proteins co-localize to discrete subnuclear foci that redistribute to sites of DNA damage under genotoxic stress [89]. BRCA1 also co-localizes with phosphorylated H2AX (γH2AX) in response to double strand breaks [90].

The central region of human BRCA1 binds strongly to negatively supercoiled plasmid DNA with native superhelical density [50] and binds with high affinity to cruciform DNA [91]. The BRCA1 cruciform DNA complex must dissociate to allow the nuclease complex to work in DNA recombinational repair of double stranded breaks. BRCA1 also acts as a scaffold for assembly of the Rad51 ATPase which is responsible for homologous recombination in somatic cells. The full-length BRCA1 protein binds strongly to supercoiled plasmid DNA and to junction DNA. The difference in affinity was on the order of 6- to 7-fold between linear and junction DNA in reactions containing physiological levels of magnesium [92]. BRCA1 230-534 binds with a higher affinity to four-way junction DNA as compared to duplex and single-stranded DNA [91]. Residues 340-554 of BRCA1 have been identified as the minimal DNA-binding region [93]. The highest affinity among the different DNA targets which mimic damaged DNA (four-way junction DNA, DNA mismatches, DNA bulges and linear DNA) was for DNA four-way junctions. To this end, a 20-fold excess of linear DNA was unable to compete off any of the BRCA1 230-534 bound to DNA molecules mimicking damaged DNA [49]. Furthermore, the loss of the BRCA1 gene prevents cell survival after exposure to DNA cross-linkers such as mitomycin C [94]. These results speak to the importance of BRCA1's ability to recognize cruciform structures.

##### HMGB family

The high mobility-group (HMG) proteins are a family of abundant and ubiquitous non-histone proteins that are known to bind to eukaryotic chromatin. The three HMG protein families comprise the (a) HMGA proteins (formerly HMGI/Y) containing A/T-hook DNA-binding motifs, (b) HMGB proteins (formerly HMG1/2) containing HMG-box domain(s), and (c) HMGN proteins (formerly HMG14/17) containing a nucleosome-binding domain [95].

HMGB proteins bind DNA in a sequence independent manner and are known to bind to certain DNA structures (four-way junctions, DNA minicircles, cis-platinated DNA, etc.) with high affinity as compared to linear DNA [96,97]. The chromatin architectural protein HMGB1 can bind with extremely high affinity to DNA structures that form DNA loops [72], while other studies have shown that the HMG box of different proteins can induce DNA bending [98-100]. The HMG box is an 80 amino acid domain found in a variety of eukaryotic chromosomal proteins and transcription factors. HMG box binding to DNA is associated with distortions in DNA structure. Members of the HMG protein family are involved in transcription [101-103] and DNA repair [57,104,105]. The HMG protein T160 was found to be co-localized with DNA replication foci [106]. The fact that all HMG box domains bind to four-way DNA junctions suggests that a common feature in the binding targets of this protein family must exist. Single HMG box domains interact exclusively with the open square form of the junction, and conditions that stabilize the stacked × structure conformation significantly weaken the HMG box DNA interaction [107]. Binding of the isolated A domain of HMGB1 protein to four-way junction DNA substrates is abolished by mutation of both Lys2 and Lys11 together to alanine, indicating that these residues play an important role in DNA binding [108].

#### Proteins involved in replication

Transient transitions from B-DNA to cruciform structures are correlated with DNA replication and transcription [109]. It has been shown that cruciforms serve as recognition signals at or near eukaryotic origins of DNA replication [110-112]. There are a large number of proteins involved in replication which bind to cruciform structures (see Table 1). We focus here primarily on the 14-3-3 protein family and MLL and WRN proteins. We will comment briefly on other systems of interest.

S16 is a structure-specific DNA-binding protein displaying preferential binding for cruciform DNA structures [113]. The AF10 protein binds cruciform DNA via a specific interaction with an AT-hook motif and is localized to the nucleus by a defined bipartite nuclear localization signal in the N-terminal region [114]. The structural maintenance of chromosomes (SMC) protein family, with members from lower and higher eukaryotes, may be divided into four subfamilies (SMC1 to SMC4) and two SMC-like protein subfamilies (SMC5 and SMC6) [115-117]. Members of this family are implicated in a large range of activities that modulate chromosome structure and organization. Smc1 and smc2 proteins have a high affinity for cruciform DNA molecules and for AT-rich DNA fragments including fragments from the scaffold-associated regions [118]. The baculovirus very late expression factor 1 (VLF-1), a member of the integrase protein family, does not bind to single and double strand structures, but it does bind (listed with increasing affinity) to Y-forks, three-way junctions and cruciform structures. This protein is involved in the processing of branched DNA molecules at the late stages of viral genome replication [119].

##### 14-3-3

The 14-3-3 protein family consists of a highly conserved and widely distributed group of dimeric proteins which occur as multiple isoforms in eukaryotes [120]. There are at least seven distinct 14-3-3 genes in vertebrates, giving rise to nine isoforms (α, β, γ, δ, ε, ζ, η, σ and τ) and at least another 20 have been identified in yeast, plants, amphibians and invertebrates [110]. A striking feature of the 14-3-3 proteins is their ability to bind a multitude of functionally diverse signaling proteins, including kinases, phosphatases, and transmembrane receptors. This plethora of proteins allows 14-3-3s to modulate a wide variety of vital regulatory processes, including mitogenic signal transduction, apoptosis and cell cycle regulation [121]. The 14-3-3 proteins are found mainly within the nucleus and are involved in eukaryotic DNA replication via binding to the cruciform DNA that forms transiently at replication origins at the onset of the S phase [122].

14-3-3 cruciform binding activity was first observed in proteins purified from sheep's brain. More recently, immunofluorescence analyses showed that 14-3-3 isoforms with cruciform-binding activity are present in HeLa cells [123]. The direct interaction with cruciform DNA was confirmed with 14-3-3 isoforms β, γ, σ, ε, and ζ [34]. 14-3-3 analogs with cruciform-specific binding are also found in yeast (Bmh1 and Bmh2) and plants (GF14) [35].

The prevalence of the 14-3-3 family proteins in all eukaryotes combined with a high degree of sequence conservation between species is indicative of their importance. Genetic studies have shown that knocking out the yeasts homologs of the 14-3-3 proteins is lethal [124]. Moreover, 14-3-3 proteins are involved in interactions with numerous transcription factors and it has been reported that several of the 14-3-3 proteins functions are associated with its cruciform binding properties.

##### Mixed lineage leukemia (MLL) protein

The MLL gene encodes a putative transcription factor with regions of homology to several other proteins including the zinc fingers and the so-called "AT-hook" DNA-binding motif of high mobility group proteins [125]. The 11q23 chromosomal translocation, found in both acute lymphoid and myeloid leukemias, results in disruption of the MLL gene. Leukemogenesis is often correlated with alternations in chromatin structure brought about by either a gain or loss in function of the regulatory factors due to their being disrupted by chromosomal translocations. The MLL gene, a target of such translocation events, forms a chimeric fusion product with a variety of partner genes [126].

The MLL AT-hook domain binds cruciform DNA, recognizing the structure rather than the sequence of the target DNA. This interaction can be antagonized both by Hoechst 33258 dye and distamycin. In a nitrocellulose protein-DNA binding assay, the MLL AT-hook domain was shown to bind to AT-rich SARs, but not to non-SAR DNA fragments [125]. MLL appears to be involved in chromatin-mediated gene regulation. In translocations involving MLL, the loss of the activation domain combined with the retention of a repression domain alters the expression of downstream target genes, thus suggesting a potential mechanism of action for MLL in leukemia [126]. AF10 translocations to the vicinity of genes other than MLL also result in myeloid leukemia. A biochemical analysis of the MLL partner gene AF10 showed that its AT-hook motif is able to bind to cruciform DNA, but not to double-stranded DNA, and that it forms a homo-tetramer *in vitro *[114].

##### WRN

The Werner syndrome protein belongs to the RecQ family of evolutionary conserved 3' → 5' DNA helicases [127]. WRN encodes a single polypeptide of 162 kDa that contains 1432 amino acids. Prokaryotes and lower eukaryotes generally have one RecQ member while higher eukaryotes possess multiple members and five homologs have been identified in human cells. All RecQ members share a conserved helicase core with one or two additional C-terminal domains, the RQC (RecQ C-terminal) and HRDC (helicase and RNaseD C-terminal) domains. These domains bind both to proteins and DNA. Eukaryotic RecQ helicases have N- and C-terminal extensions that are involved in protein-protein interactions and have been postulated to lend unique functional characteristics to these proteins [55,128]. WRN has been shown to bind at replication fork junctions and to Holliday junction structures. Binding to junction DNA is highly specific because little or no WRN binding is visualized at other sites along these substrates [129]. Upon binding to DNA, WRN assembles into a large complex composed of four monomers.

#### Cruciform binding proteins and disease

The recognition of DNA junctions and cruciform structures is critical for genomic stability and for the regulation of basic cellular processes. The resolution of Holliday junctions and long cruciforms is necessary for genomic stability where the dysregulation of these proteins can lead to DNA translocations, deletions, loss of genomics stability and carcinogenesis. The large numbers of proteins which bind to these DNA structures work together to keep the genome intact. We believe that the formation of cruciform structures serves as a marker for the proper timing and initiation of some very basic biological processes. The mutations and epigenetic modifications that alter the propensity for cruciform formation can have drastic consequences for cellular processes. Thus, it is unsurprising that the dysregulation of cruciform binding proteins is often associated with the pathology of disease.

As stated above, the cruciform binding proteins including p53, BRCA1, WRN and the proto-oncogenes DEK, MLL and HMG are also associated with cancer development and/or progression. Some of these proteins play such important roles that their mutation and/or inactivation result in severe genomic instability and sometimes lethality. For example, Brca1 -/- mouse embryonic stem cells show spontaneous chromosome breakage, profound genomic instability and hypersensitivity to a variety of damaging agents (e.g. γ radiation) all of which suggests a defect in DNA repair. The connection between the BRCA1 mutation and breast cancer is well known. P53's transcriptional regulation is fine-tuned by its timely binding to promoter elements. The formation of a cruciform structure in p53 recognition elements may be an important determinant of p53 transcription activity.

The dHMGI(Y) family of "high mobility group" non-histone proteins comprises architectural transcription factors whose over expression is highly correlated with carcinogenesis, increased malignancy and metastatic potential of tumors *in vivo *[95]. 14-3-3 proteins are related to several diseases, including cancer, Alzeheimer's disease, the neurological Miller Dieker and Spinocerebellar ataxia type 1 diseases, and spongiform encephalopathy. The deletion of 14-3-3σ in human colorectal cancer cells leads to the loss of the DNA damage checkpoint control [130]. The human DEK protein was discovered as a fusion with a nuclear pore protein in a subset of patients with acute myeloid leukemia. It was also identified as an autoantigen in a relatively high percentage of patients with autoimmune diseases. In addition, DEK mRNA levels are higher in transcriptionally active and proliferating cells than in resting cells, and elevated mRNA levels are found in several transformed and cancer cells [6,7]. Werner syndrome is an autosomal recessive disorder characterized by features of premature aging and a high incidence of uncommon cancers [127]. The Werner syndrome protein (WRN) plays central roles in maintaining the genomic stability of organisms [131]. Individuals harboring mutations in WRN have a rare, autosomal recessive genetic disorder manifested by early onset of symptoms characteristic of aged individuals.

## Conclusions

**Figure 5:**
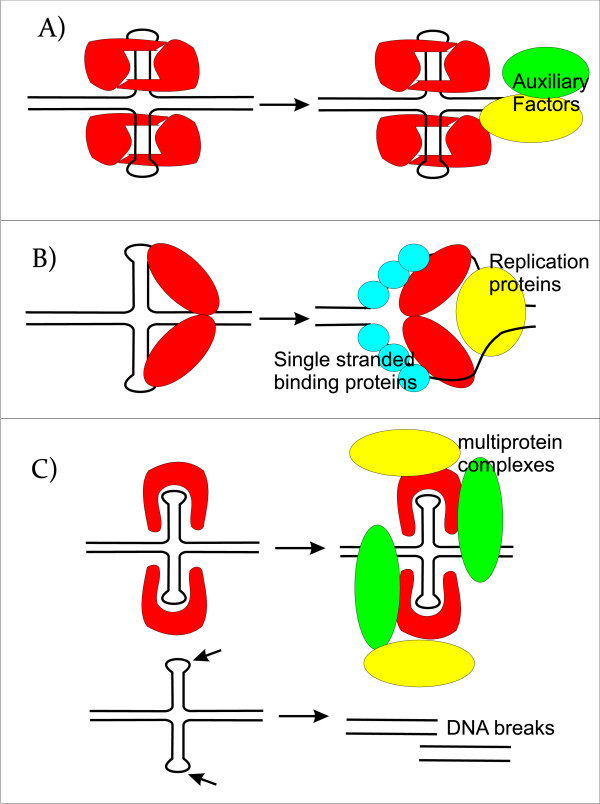
**Scheme of the putative mechanistic roles of cruciform binding proteins in transcription, DNA replication, and DNA repair**. A) A model for the structure-specific binding of transcription factors to a cognate palindrome-type cruciform implicated in transcription. The equilibrium between classic B-DNA and the higher order cruciform favors duplex DNA, but, when cruciform binding proteins are present, they either preferentially bind to and stabilize the cruciform or bind to the classic form and convert it to the cruciform. This interaction results in both an initial melting of the DNA region covered by transcription factor and an extension of the melt region in both directions. The melting region continues to extend in response to the needs of the active transcription machinery. B) A model for the initiation of replication enhanced by extrusion to a cruciform structure. Dimeric cruciform binding proteins interact with and stabilize the cruciform structure. The replisome is assembled concomitantly and is assumed to include polymerases, single-strand binding proteins and helicases. C) Model for the influence of cruciform binding proteins on DNA structure in DNA damage regulation. Naked cruciforms are sensitive to DNA damage and are covered by proteins in order to protect these sequences from being cleaved. In these cases, a deficiency in cruciform binding proteins can lead to DNA breaks. Here, cruciform-DNA complexes can also serve as scaffolds to recruit the DNA damage machinery.

Cruciform structures are fundamentally important for a wide range of biological processes, including DNA transcription, replication, recombination, control of gene expression and genome organization. The putative mechanistic roles of cruciform binding proteins in transcription, DNA replication, and DNA repair are shown in Figure 5. Alternative DNA structures, including cruciforms, are often formed at sites of negatively supercoiled DNA by perfect or imperfect inverted repeats of 6 or more nucleotides. Longer DNA palindromes present a threat to genomic stability as they are recognized by junction-resolving enzymes. Shorter palindromic sequences are essential for basic processes like DNA replication and transcription. The presence of cruciform structures may also play an important role in epigenetics, such that cruciform structures are protected from DNA methylation. For example, the Dam methylase is not able to modify its GATC target site when it occurs in a cruciform or hairpin conformation. The center of a long perfect palindrome located in bacteriophage lambda has also been shown to be methylation-resistant *in vivo *[40]. Moreover, the centers of long palindromes are hypo-methylated as compared to identical sequences in non-palindromic conformations [40]. To this end, transient cruciforms can directly influence DNA methylation and therefore provide another layer for regulation of the DNA code. Proteins that bind to cruciforms can be divided into several categories. In addition to a well defined group of junction-resolving enzymes, we have classified cruciform binding proteins into groups involved in transcription and DNA repair (PARP, BRCA1, p53, 14-3-3), chromatin-associated proteins (DEK, BRCA1, HMG protein family, topoisomerases), and proteins involved in replication (MLL, WRN, 14-3-3, helicases) (see Table 1). Within these groups are proteins indispensable for cell viability, as well as tumor suppressors, proto-oncogenes and DNA remodeling proteins. Similarly, triplet repeat expansion, a phenomenon important in several genetic diseases, including Friedreich's ataxia, cardiomyopathy, myotonic dystrophy type I and other neurological disorders, can change the spectrum of cruciform binding proteins. Lastly, single nucleotide polymorphisms and/or insertion/deletion mutations at inverted repeats located in promoter sites can also influence cruciform formation, which might be manifested through altered gene regulation. A deeper understanding of the processes related to the formation and function of alternative DNA structures will be an important component to consider in the post-genomic era.

## Abbreviations

scDNA: supercoiled DNA.

## Competing interests

The authors declare that they have no competing interests.

## Authors' contributions

All authors contributed to this review. VB made ready Figures 1, 4 and 5. RL made ready Figures 2 and 3. All authors read and approved the final manuscript.

## References

[B1] Smith GR Meeting DNA palindromes head-to-head Genes Dev 2008 22 19 2612 2620 10.1101/gad.1724708 18832065 PMC2751023

[B2] Palecek E Local supercoil-stabilized DNA structures Crit Rev Biochem Mol Biol 1991 26 151 226 10.3109/10409239109081126 1914495

[B3] van Holde K Zlatanova J Unusual DNA structures, chromatin and transcription Bioessays 1994 16 1 59 68 10.1002/bies.950160110 8141807

[B4] Krasilnikov AS Podtelezhnikov A Vologodskii A Mirkin SM Large-scale effects of transcriptional DNA supercoiling in vivo J Mol Biol 1999 292 5 1149 1160 10.1006/jmbi.1999.3117 10512709

[B5] Mikheikin AL Lushnikov AY Lyubchenko YL Effect of DNA supercoiling on the geometry of holliday junctions Biochemistry 2006 45 43 12998 13006 10.1021/bi061002k 17059216 PMC1646289

[B6] Limanskaia O Limanskii AP Distribution of potentially hairpin-loop structures in the genome of bovine retroviruses Vopr Virusol 2009 54 4 27 32 19708552

[B7] Werbowy K Cieslinski H Kur J Characterization of a cryptic plasmid pSFKW33 from Shewanella sp. 33B Plasmid 2009 62 1 44 49 10.1016/j.plasmid.2009.03.003 19336243

[B8] Pearson CE Zorbas H Price GB Zannis-Hadjopoulos M Inverted repeats, stem-loops, and cruciforms: significance for initiation of DNA replication J Cell Biochem 1996 63 1 1 22 10.1002/(SICI)1097-4644(199610)63:1<1::AID-JCB1>3.0.CO;2-3 8891900

[B9] Aranda A Perez-Ortin JE Benham CJ Del Olmo ML Analysis of the structure of a natural alternating d(TA)n sequence in yeast chromatin Yeast 1997 13 4 313 326 10.1002/(SICI)1097-0061(19970330)13:4<313::AID-YEA93>3.0.CO;2-8 9133735

[B10] Bates AD Maxwell A DNA Topology 2005 second Oxford: Oxford University Press

[B11] Mani P Yadav VK Das SK Chowdhury S Genome-wide analyses of recombination prone regions predict role of DNA structural motif in recombination PLoS One 2009 4 2 e4399 10.1371/journal.pone.0004399 19198658 PMC2635932

[B12] Lin CT Lyu YL Liu LF A cruciform-dumbbell model for inverted dimer formation mediated by inverted repeats Nucleic Acids Res 1997 25 15 3009 3016 10.1093/nar/25.15.3009 9224600 PMC146860

[B13] Kim E Deppert W The complex interactions of p53 with target DNA: we learn as we go Biochem Cell Biol 2003 81 3 141 150 10.1139/o03-046 12897847

[B14] Drolet M Growth inhibition mediated by excess negative supercoiling: the interplay between transcription elongation, R-loop formation and DNA topology Mol Microbiol 2006 59 3 723 730 10.1111/j.1365-2958.2005.05006.x 16420346

[B15] Peter BJ Arsuaga J Breier AM Khodursky AB Brown PO Cozzarelli NR Genomic transcriptional response to loss of chromosomal supercoiling in Escherichia coli Genome Biol 2004 5 11 R87 10.1186/gb-2004-5-11-r87 15535863 PMC545778

[B16] Mazur SJ Sakaguchi K Appella E Wang XW Harris CC Bohr VA Preferential binding of tumor suppressor p53 to positively or negatively supercoiled DNA involves the C-terminal domain J Mol Biol 1999 292 2 241 249 10.1006/jmbi.1999.3064 10493872

[B17] Brazdova M Palecek J Cherny DI Billova S Fojta M Pecinka P Vojtesek B Jovin TM Palecek E Role of tumor suppressor p53 domains in selective binding to supercoiled DNA Nucleic Acids Res 2002 30 22 4966 4974 10.1093/nar/gkf616 12434001 PMC137164

[B18] Campos J Gonzalez-Quintela A Quinteiro C Gude F Perez LF Torre JA Vidal C The -159C/T polymorphism in the promoter region of the CD14 gene is associated with advanced liver disease and higher serum levels of acute-phase proteins in heavy drinkers Alcohol Clin Exp Res 2005 29 7 1206 1213 10.1097/01.ALC.0000171977.25531.7A 16046876

[B19] Peter BJ Ullsperger C Hiasa H Marians KJ Cozzarelli NR The structure of supercoiled intermediates in DNA replication Cell 1998 94 6 819 827 10.1016/S0092-8674(00)81740-7 9753328

[B20] Vologodskii AV Cozzarelli NR Conformational and thermodynamic properties of supercoiled DNA Annu Rev Biophys Biomol Struct 1994 23 609 643 10.1146/annurev.bb.23.060194.003141 7919794

[B21] Vologodskii A Cozzarelli NR Effect of supercoiling on the juxtaposition and relative orientation of DNA sites Biophys J 1996 70 6 2548 2556 10.1016/S0006-3495(96)79826-0 8744294 PMC1225236

[B22] Lyubchenko YL DNA structure and dynamics: an atomic force microscopy study Cell Biochem Biophys 2004 41 1 75 98 10.1385/CBB:41:1:075 15371641

[B23] Kurahashi H Inagaki H Yamada K Ohye T Taniguchi M Emanuel BS Toda T Cruciform DNA structure underlies the etiology for palindrome-mediated human chromosomal translocations J Biol Chem 2004 279 34 35377 35383 10.1074/jbc.M400354200 15208332 PMC2810964

[B24] Shlyakhtenko LS Potaman VN Sinden RR Lyubchenko YL Structure and dynamics of supercoil-stabilized DNA cruciforms J Mol Biol 1998 280 1 61 72 10.1006/jmbi.1998.1855 9653031

[B25] Declais AC Lilley DM New insight into the recognition of branched DNA structure by junction-resolving enzymes Curr Opin Struct Biol 2008 18 1 86 95 10.1016/j.sbi.2007.11.001 18160275

[B26] Tolmasky ME Colloms S Blakely G Sherratt DJ Stability by multimer resolution of pJHCMW1 is due to the Tn1331 resolvase and not to the Escherichia coli Xer system Microbiology 2000 146 Pt 3 581 589 10746761 10.1099/00221287-146-3-581

[B27] Shlyakhtenko LS Hsieh P Grigoriev M Potaman VN Sinden RR Lyubchenko YL A cruciform structural transition provides a molecular switch for chromosome structure and dynamics J Mol Biol 2000 296 5 1169 1173 10.1006/jmbi.2000.3542 10698623

[B28] Panayotatos N Fontaine A A native cruciform DNA structure probed in bacteria by recombinant T7 endonuclease J Biol Chem 1987 262 23 11364 11368 3038915

[B29] Noirot P Bargonetti J Novick RP Initiation of rolling-circle replication in pT181 plasmid: initiator protein enhances cruciform extrusion at the origin Proc Natl Acad Sci USA 1990 87 21 8560 8564 10.1073/pnas.87.21.8560 2236066 PMC54996

[B30] Yamaguchi K Yamaguchi M The replication origin of pSC101: the nucleotide sequence and replication functions of the ori region Gene 1984 29 1-2 211 219 10.1016/0378-1119(84)90181-1 6092223

[B31] Yahyaoui W Callejo M Price GB Zannis-Hadjopoulos M Deletion of the cruciform binding domain in CBP/14-3-3 displays reduced origin binding and initiation of DNA replication in budding yeast BMC Mol Biol 2007 8 27 10.1186/1471-2199-8-27 17430600 PMC1865385

[B32] Bell D Sabloff M Zannis-Hadjopoulos M Price G Anti-cruciform DNA affinity purification of active mammalian origins of replication Biochim Biophys Acta 1991 1089 3 299 308 1859833 10.1016/0167-4781(91)90169-m

[B33] Zannis-Hadjopoulos M Frappier L Khoury M Price GB Effect of anti-cruciform DNA monoclonal antibodies on DNA replication Embo J 1988 7 6 1837 1844 3169006 10.1002/j.1460-2075.1988.tb03016.x PMC457176

[B34] Alvarez D Novac O Callejo M Ruiz MT Price GB Zannis-Hadjopoulos M 14-3-3sigma is a cruciform DNA binding protein and associates in vivo with origins of DNA replication J Cell Biochem 2002 87 2 194 207 10.1002/jcb.10294 12244572

[B35] Callejo M Alvarez D Price GB Zannis-Hadjopoulos M The 14-3-3 protein homologues from Saccharomyces cerevisiae, Bmh1p and Bmh2p, have cruciform DNA-binding activity and associate in vivo with ARS307 J Biol Chem 2002 277 41 38416 38423 10.1074/jbc.M202050200 12167636

[B36] Haniford DB Pulleyblank DE Transition of a cloned d(AT)n-d(AT)n tract to a cruciform in vivo Nucleic Acids Res 1985 13 12 4343 4363 10.1093/nar/13.12.4343 4011446 PMC321792

[B37] Hanke JH Hambor JE Kavathas P Repetitive Alu elements form a cruciform structure that regulates the function of the human CD8 alpha T cell-specific enhancer J Mol Biol 1995 246 1 63 73 10.1006/jmbi.1994.0066 7853405

[B38] Dayn A Malkhosyan S Mirkin SM Transcriptionally driven cruciform formation in vivo Nucleic Acids Res 1992 20 22 5991 5997 10.1093/nar/20.22.5991 1461732 PMC334465

[B39] Xu J De Zhu J Ni M Wan F Gu JR The ATF/CREB site is the key element for transcription of the human RNA methyltransferase like 1(RNMTL1) gene, a newly discovered 17p13.3 gene Cell Res 2002 12 3-4 177 197 10.1038/sj.cr.7290124 12296377

[B40] Allers T Leach DR DNA palindromes adopt a methylation-resistant conformation that is consistent with DNA cruciform or hairpin formation in vivo J Mol Biol 1995 252 1 70 85 10.1006/jmbi.1994.0476 7666435

[B41] Harada S Uchida M Shimizu N Episomal high copy number maintenance of hairpin-capped DNA bearing a replication initiation region in human cells J Biol Chem 2009 284 36 24320 24327 10.1074/jbc.M109.008128 19617622 PMC2782025

[B42] Cote AG Lewis SM Mus81-dependent double-strand DNA breaks at in vivo-generated cruciform structures in S. cerevisiae Mol Cell 2008 31 6 800 812 10.1016/j.molcel.2008.08.025 18922464

[B43] Lilley DM White MF The junction-resolving enzymes Nat Rev Mol Cell Biol 2001 2 6 433 443 10.1038/35073057 11389467

[B44] Aravind L Makarova KS Koonin EV SURVEY AND SUMMARY: holliday junction resolvases and related nucleases: identification of new families, phyletic distribution and evolutionary trajectories Nucleic Acids Res 2000 28 18 3417 3432 10.1093/nar/28.18.3417 10982859 PMC110722

[B45] Khuu PA Voth AR Hays FA Ho PS The stacked-X DNA Holliday junction and protein recognition J Mol Recognit 2006 19 3 234 242 10.1002/jmr.765 16575941 PMC4537160

[B46] Lilley DM Structures of helical junctions in nucleic acids Q Rev Biophys 2000 33 2 109 159 10.1017/S0033583500003590 11131562

[B47] Stefanovsky VY Moss T The cruciform DNA mobility shift assay: a tool to study proteins that recognize bent DNA Methods Mol Biol 2009 543 537 546 10.1007/978-1-60327-015-1_31 19378185

[B48] Mazina OM Rossi MJ Thomaa NH Mazin AV Interactions of human rad54 protein with branched DNA molecules J Biol Chem 2007 282 29 21068 21080 10.1074/jbc.M701992200 17545145

[B49] Naseem R Webb M Analysis of the DNA binding activity of BRCA1 and its modulation by the tumour suppressor p53 PLoS ONE 2008 3 6 e2336 10.1371/journal.pone.0002336 18545657 PMC2396507

[B50] Brazda V Jagelska EB Liao JC Arrowsmith CH The central region of BRCA1 binds preferentially to supercoiled DNA J Biomol Struct Dyn 2009 27 1 97 104 19492866 10.1080/07391102.2009.10507299

[B51] Chasovskikh S Dimtchev A Smulson M Dritschilo A DNA transitions induced by binding of PARP-1 to cruciform structures in supercoiled plasmids Cytometry A 2005 68 1 21 27 16200639 10.1002/cyto.a.20187

[B52] Poulet A Buisson R Faivre-Moskalenko C Koelblen M Amiard S Montel F Cuesta-Lopez S Bornet O Guerlesquin F Godet T TRF2 promotes, remodels and protects telomeric Holliday junctions Embo J 2009 28 6 641 651 10.1038/emboj.2009.11 19197240 PMC2666026

[B53] Shiba T Iwasaki H Nakata A Shinagawa H SOS-inducible DNA repair proteins, RuvA and RuvB, of Escherichia coli: functional interactions between RuvA and RuvB for ATP hydrolysis and renaturation of the cruciform structure in supercoiled DNA Proc Natl Acad Sci USA 1991 88 19 8445 8449 10.1073/pnas.88.19.8445 1833759 PMC52525

[B54] Iwasaki H Takahagi M Nakata A Shinagawa H Escherichia coli RuvA and RuvB proteins specifically interact with Holliday junctions and promote branch migration Genes Dev 1992 6 11 2214 2220 10.1101/gad.6.11.2214 1427081

[B55] van Brabant AJ Stan R Ellis NA DNA helicases, genomic instability, and human genetic disease Annu Rev Genomics Hum Genet 2000 1 409 459 10.1146/annurev.genom.1.1.409 11701636

[B56] Wakasugi M Reardon JT Sancar A The non-catalytic function of XPG protein during dual incision in human nucleotide excision repair J Biol Chem 1997 272 25 16030 16034 10.1074/jbc.272.25.16030 9188507

[B57] Stros M Bacikova A Polanska E Stokrova J Strauss F HMGB1 interacts with human topoisomerase IIalpha and stimulates its catalytic activity Nucleic Acids Res 2007 35 15 5001 5013 10.1093/nar/gkm525 17636313 PMC1976466

[B58] Klungland H Andersen O Kisen G Alestrom P Tora L Estrogen receptor binds to the salmon GnRH gene in a region with long palindromic sequences Mol Cell Endocrinol 1993 95 1-2 147 154 10.1016/0303-7207(93)90040-Q 8243805

[B59] Benjamin RC Gill DM Poly(ADP-ribose) synthesis in vitro programmed by damaged DNA. A comparison of DNA molecules containing different types of strand breaks J Biol Chem 1980 255 21 10502 10508 6253477

[B60] Rouleau M Aubin RA Poirier GG Poly(ADP-ribosyl)ated chromatin domains: access granted J Cell Sci 2004 117 Pt 6 815 825 14963022 10.1242/jcs.01080

[B61] Tulin A Chinenov Y Spradling A Regulation of chromatin structure and gene activity by poly(ADP-ribose) polymerases Curr Top Dev Biol 2003 56 55 83 14584726 10.1016/s0070-2153(03)01007-x

[B62] Soldatenkov VA Chasovskikh S Potaman VN Trofimova I Smulson ME Dritschilo A Transcriptional repression by binding of poly(ADP-ribose) polymerase to promoter sequences J Biol Chem 2002 277 1 665 670 11684688 10.1074/jbc.M108551200

[B63] Lonskaya I Potaman VN Shlyakhtenko LS Oussatcheva EA Lyubchenko YL Soldatenkov VA Regulation of poly(ADP-ribose) polymerase-1 by DNA structure-specific binding J Biol Chem 2005 280 17 17076 17083 10.1074/jbc.M413483200 15737996

[B64] Dey A Verma CS Lane DP Updates on p53: modulation of p53 degradation as a therapeutic approach Br J Cancer 2008 98 1 4 8 10.1038/sj.bjc.6604098 18182973 PMC2359710

[B65] Kim E Rohaly G Heinrichs S Gimnopoulos D Meissner H Deppert W Influence of promoter DNA topology on sequence-specific DNA binding and transactivation by tumor suppressor p53 Oncogene 1999 18 51 7310 7318 10.1038/sj.onc.1203139 10602486

[B66] Brazda V Jagelska EB Fojta M Palecek E Searching for target sequences by p53 protein is influenced by DNA length Biochem Biophys Res Commun 2006 341 2 470 477 10.1016/j.bbrc.2005.12.202 16426567

[B67] Brazda V Muller P Brozkova K Vojtesek B Restoring wild-type conformation and DNA-binding activity of mutant p53 is insufficient for restoration of transcriptional activity Biochem Biophys Res Commun 2006 351 2 499 506 10.1016/j.bbrc.2006.10.065 17070499

[B68] Palecek E Vlk D Stankova V Brazda V Vojtesek B Hupp TR Schaper A Jovin TM Tumor suppressor protein p53 binds preferentially to supercoiled DNA Oncogene 1997 15 18 2201 2209 10.1038/sj.onc.1201398 9393978

[B69] Brazda V Palecek J Pospisilova S Vojtesek B Palecek E Specific modulation of p53 binding to consensus sequence within supercoiled DNA by monoclonal antibodies Biochem Biophys Res Commun 2000 267 3 934 939 10.1006/bbrc.1999.2056 10673394

[B70] Degtyareva N Subramanian D Griffith JD Analysis of the binding of p53 to DNAs containing mismatched and bulged bases J Biol Chem 2001 276 12 8778 8784 10.1074/jbc.M006795200 11124254

[B71] Nagaich AK Appella E Harrington RE DNA bending is essential for the site-specific recognition of DNA response elements by the DNA binding domain of the tumor suppressor protein p53 J Biol Chem 1997 272 23 14842 14849 10.1074/jbc.272.23.14842 9169453

[B72] Stros M Muselikova-Polanska E Pospisilova S Strauss F High-affinity binding of tumor-suppressor protein p53 and HMGB1 to hemicatenated DNA loops Biochemistry 2004 43 22 7215 7225 10.1021/bi049928k 15170359

[B73] Subramanian D Griffith JD Modulation of p53 binding to Holliday junctions and 3-cytosine bulges by phosphorylation events Biochemistry 2005 44 7 2536 2544 10.1021/bi048700u 15709766

[B74] Griffith JD Comeau L Rosenfield S Stansel RM Bianchi A Moss H de Lange T Mammalian telomeres end in a large duplex loop Cell 1999 97 4 503 514 10.1016/S0092-8674(00)80760-6 10338214

[B75] Jagelska EB Brazda V Pecinka P Palecek E Fojta M DNA topology influences p53 sequence-specific DNA binding through structural transitions within the target sites Biochem J 2008 412 1 57 63 10.1042/BJ20071648 18271758

[B76] Jagelska EB Pivonkova H Fojta M Brazda V The potential of the cruciform structure formation as an important factor influencing p53 sequence-specific binding to natural DNA targets Biochem Biophys Res Commun 2010 391 3 1409 1414 10.1016/j.bbrc.2009.12.076 20026061

[B77] Hede MS Petersen RL Frohlich RF Kruger D Andersen FF Andersen AH Knudsen BR Resolution of Holliday junction substrates by human topoisomerase I J Mol Biol 2007 365 4 1076 1092 10.1016/j.jmb.2006.10.050 17101150

[B78] Lee GE Kim JH Chung IK Topoisomerase II-mediated DNA cleavage on the cruciform structure formed within the 5'upstream region of the human beta-globin gene Mol Cells 1998 8 4 424 430 9749529

[B79] Heyer WD Li X Rolfsmeier M Zhang XP Rad54: the Swiss Army knife of homologous recombination? Nucleic Acids Res 2006 34 15 4115 4125 10.1093/nar/gkl481 16935872 PMC1616967

[B80] Bugreev DV Mazina OM Mazin AV Rad54 protein promotes branch migration of Holliday junctions Nature 2006 442 7102 590 593 10.1038/nature04889 16862129

[B81] Modesti M Budzowska M Baldeyron C Demmers JA Ghirlando R Kanaar R RAD51AP1 is a structure-specific DNA binding protein that stimulates joint molecule formation during RAD51-mediated homologous recombination Mol Cell 2007 28 3 468 481 10.1016/j.molcel.2007.08.025 17996710

[B82] Kappes F Burger K Baack M Fackelmayer FO Gruss C Subcellular localization of the human proto-oncogene protein DEK J Biol Chem 2001 276 28 26317 26323 10.1074/jbc.M100162200 11333257

[B83] Waldmann T Scholten I Kappes F Hu HG Knippers R The DEK protein--an abundant and ubiquitous constituent of mammalian chromatin Gene 2004 343 1 1 9 10.1016/j.gene.2004.08.029 15563827

[B84] Waldmann T Baack M Richter N Gruss C Structure-specific binding of the proto-oncogene protein DEK to DNA Nucleic Acids Res 2003 31 23 7003 7010 10.1093/nar/gkg864 14627833 PMC290247

[B85] Alexiadis V Waldmann T Andersen J Mann M Knippers R Gruss C The protein encoded by the proto-oncogene DEK changes the topology of chromatin and reduces the efficiency of DNA replication in a chromatin-specific manner Genes Dev 2000 14 11 1308 1312 10837023 PMC316669

[B86] Kappes F Damoc C Knippers R Przybylski M Pinna LA Gruss C Phosphorylation by protein kinase CK2 changes the DNA binding properties of the human chromatin protein DEK Mol Cell Biol 2004 24 13 6011 6020 10.1128/MCB.24.13.6011-6020.2004 15199154 PMC480878

[B87] Kappes F Scholten I Richter N Gruss C Waldmann T Functional domains of the ubiquitous chromatin protein DEK Mol Cell Biol 2004 24 13 6000 6010 10.1128/MCB.24.13.6000-6010.2004 15199153 PMC480879

[B88] Bohm F Kappes F Scholten I Richter N Matsuo H Knippers R Waldmann T The SAF-box domain of chromatin protein DEK Nucleic Acids Res 2005 33 3 1101 1110 10.1093/nar/gki258 15722484 PMC549417

[B89] Scully R Chen J Ochs RL Keegan K Hoekstra M Feunteun J Livingston DM Dynamic changes of BRCA1 subnuclear location and phosphorylation state are initiated by DNA damage Cell 1997 90 3 425 435 10.1016/S0092-8674(00)80503-6 9267023

[B90] Paull TT Rogakou EP Yamazaki V Kirchgessner CU Gellert M Bonner WM A critical role for histone H2AX in recruitment of repair factors to nuclear foci after DNA damage Curr Biol 2000 10 15 886 895 10.1016/S0960-9822(00)00610-2 10959836

[B91] Sturdy A Naseem R Webb M Purification and characterisation of a soluble N-terminal fragment of the breast cancer susceptibility protein BRCA1 J Mol Biol 2004 340 3 469 475 10.1016/j.jmb.2004.05.005 15210348

[B92] Paull TT Cortez D Bowers B Elledge SJ Gellert M Direct DNA binding by Brca1 Proc Natl Acad Sci USA 2001 98 11 6086 6091 10.1073/pnas.111125998 11353843 PMC33426

[B93] Naseem R Sturdy A Finch D Jowitt T Webb M Mapping and conformational characterization of the DNA-binding region of the breast cancer susceptibility protein BRCA1 Biochem J 2006 395 3 529 535 10.1042/BJ20051646 16460311 PMC1462700

[B94] De la Torre C Pincheira J Lopez-Saez JF Human syndromes with genomic instability and multiprotein machines that repair DNA double-strand breaks Histol Histopathol 2003 18 1 225 243 12507302 10.14670/HH-18.225

[B95] Banks GC Li Y Reeves R Differential in vivo modifications of the HMGI(Y) nonhistone chromatin proteins modulate nucleosome and DNA interactions Biochemistry 2000 39 28 8333 8346 10.1021/bi000378+ 10889043

[B96] Grasser KD Teo SH Lee KB Broadhurst RW Rees C Hardman CH Thomas JO DNA-binding properties of the tandem HMG boxes of high-mobility-group protein 1 (HMG1) Eur J Biochem 1998 253 3 787 795 10.1046/j.1432-1327.1998.2530787.x 9654080

[B97] Agresti A Bianchi ME HMGB proteins and gene expression Curr Opin Genet Dev 2003 13 2 170 178 10.1016/S0959-437X(03)00023-6 12672494

[B98] Deckert J Khalaf RA Hwang SM Zitomer RS Characterization of the DNA binding and bending HMG domain of the yeast hypoxic repressor Rox1 Nucleic Acids Res 1999 27 17 3518 3526 10.1093/nar/27.17.3518 10446242 PMC148596

[B99] Phillips NB Nikolskaya T Jancso-Radek A Ittah V Jiang F Singh R Haas E Weiss MA Sry-directed sex reversal in transgenic mice is robust with respect to enhanced DNA bending: comparison of human and murine HMG boxes Biochemistry 2004 43 22 7066 7081 10.1021/bi049920a 15170344

[B100] Dragan AI Read CM Makeyeva EN Milgotina EI Churchill ME Crane-Robinson C Privalov PL DNA binding and bending by HMG boxes: energetic determinants of specificity J Mol Biol 2004 343 2 371 393 10.1016/j.jmb.2004.08.035 15451667

[B101] Stros M Polanska E Struncova S Pospisilova S HMGB1 and HMGB2 proteins up-regulate cellular expression of human topoisomerase IIalpha Nucleic Acids Res 2009 37 7 2070 2086 10.1093/nar/gkp067 19223331 PMC2673423

[B102] Stefanovsky VY Langlois F Bazett-Jones D Pelletier G Moss T ERK modulates DNA bending and enhancesome structure by phosphorylating HMG1-boxes 1 and 2 of the RNA polymerase I transcription factor UBF Biochemistry 2006 45 11 3626 3634 10.1021/bi051782h 16533045

[B103] Harrer M Luhrs H Bustin M Scheer U Hock R Dynamic interaction of HMGA1a proteins with chromatin J Cell Sci 2004 117 Pt 16 3459 3471 15213251 10.1242/jcs.01160

[B104] Boulikas T Evolutionary consequences of nonrandom damage and repair of chromatin domains J Mol Evol 1992 35 2 156 180 1501255 10.1007/BF00183227

[B105] Kamashev D Balandina A Rouviere-Yaniv J The binding motif recognized by HU on both nicked and cruciform DNA Embo J 1999 18 19 5434 5444 10.1093/emboj/18.19.5434 10508175 PMC1171612

[B106] Hertel L De Andrea M Bellomo G Santoro P Landolfo S Gariglio M The HMG protein T160 colocalizes with DNA replication foci and is down-regulated during cell differentiation Exp Cell Res 1999 250 2 313 328 10.1006/excr.1999.4495 10413586

[B107] JR P Norman DG Bramham J Bianchi ME Lilley DM HMG box proteins bind to four-way DNA junctions in their open conformation Embo J 1998 17 3 817 826 10.1093/emboj/17.3.817 9451006 PMC1170430

[B108] Assenberg R Webb M Connolly E Stott K Watson M Hobbs J Thomas JO A critical role in structure-specific DNA binding for the acetylatable lysine residues in HMGB1 Biochem J 2008 411 3 553 561 10.1042/BJ20071613 18241198

[B109] Pearson CE Zorbas H Price GB Zannis-Hadjopoulos M Inverted repeats, stem-loops, and cruciforms: significance for initiation of DNA replication J Cell Biochem 1996 63 1 1 22 10.1002/(SICI)1097-4644(199610)63:1<1::AID-JCB1>3.0.CO;2-3 8891900

[B110] Zannis-Hadjopoulos M Yahyaoui W Callejo M 14-3-3 cruciform-binding proteins as regulators of eukaryotic DNA replication Trends Biochem Sci 2008 33 1 44 50 10.1016/j.tibs.2007.09.012 18054234

[B111] Kim E Lane CE Curtis BA Kozera C Bowman S Archibald JM Complete sequence and analysis of the mitochondrial genome of Hemiselmis andersenii CCMP644 (Cryptophyceae) BMC Genomics 2008 9 215 10.1186/1471-2164-9-215 18474103 PMC2397417

[B112] Omberg L Meyerson JR Kobayashi K Drury LS Diffley JF Alter O Global effects of DNA replication and DNA replication origin activity on eukaryotic gene expression Mol Syst Biol 2009 5 312 19888207 10.1038/msb.2009.70 PMC2779084

[B113] Bonnefoy E The ribosomal S16 protein of Escherichia coli displaying a DNA-nicking activity binds to cruciform DNA Eur J Biochem 1997 247 3 852 859 10.1111/j.1432-1033.1997.t01-1-00852.x 9288907

[B114] Linder B Newman R Jones LK Debernardi S Young BD Freemont P Verrijzer CP Saha V Biochemical analyses of the AF10 protein: the extended LAP/PHD-finger mediates oligomerisation J Mol Biol 2000 299 2 369 378 10.1006/jmbi.2000.3766 10860745

[B115] Peterson CL The SMC family: novel motor proteins for chromosome condensation? Cell 1994 79 3 389 392 10.1016/0092-8674(94)90247-X 7954805

[B116] Palecek J Vidot S Feng M Doherty AJ Lehmann AR The Smc5-Smc6 DNA repair complex. bridging of the Smc5-Smc6 heads by the KLEISIN, Nse4, and non-Kleisin subunits J Biol Chem 2006 281 48 36952 36959 10.1074/jbc.M608004200 17005570

[B117] Hirano T SMC proteins and chromosome mechanics: from bacteria to humans Philos Trans R Soc Lond B Biol Sci 2005 360 1455 507 514 10.1098/rstb.2004.1606 15897176 PMC1569466

[B118] Akhmedov AT Frei C Tsai-Pflugfelder M Kemper B Gasser SM Jessberger R Structural maintenance of chromosomes protein C-terminal domains bind preferentially to DNA with secondary structure J Biol Chem 1998 273 37 24088 24094 10.1074/jbc.273.37.24088 9727028

[B119] Mikhailov VS Rohrmann GF Binding of the baculovirus very late expression factor 1 (VLF-1) to different DNA structures BMC Mol Biol 2002 3 14 10.1186/1471-2199-3-14 12350233 PMC130038

[B120] Aitken A 14-3-3 proteins: a historic overview Semin Cancer Biol 2006 16 3 162 172 10.1016/j.semcancer.2006.03.005 16678438

[B121] Fu H Subramanian RR Masters SC 14-3-3 proteins: structure, function, and regulation Annu Rev Pharmacol Toxicol 2000 40 617 647 10.1146/annurev.pharmtox.40.1.617 10836149

[B122] Zannis-Hadjopoulos M Sibani S Price GB Eucaryotic replication origin binding proteins Front Biosci 2004 9 2133 2143 10.2741/1369 15353275

[B123] Todd A Cossons N Aitken A Price GB Zannis-Hadjopoulos M Human cruciform binding protein belongs to the 14-3-3 family Biochemistry 1998 37 40 14317 14325 10.1021/bi980768k 9760269

[B124] van Heusden GP van der Zanden AL Ferl RJ Steensma HY Four Arabidopsis thaliana 14-3-3 protein isoforms can complement the lethal yeast bmh1 bmh2 double disruption FEBS Lett 1996 391 3 252 256 10.1016/0014-5793(96)00746-6 8764984

[B125] Broeker PL Harden A Rowley JD Zeleznik-Le N The mixed lineage leukemia (MLL) protein involved in 11q23 translocations contains a domain that binds cruciform DNA and scaffold attachment region (SAR) DNA Curr Top Microbiol Immunol 1996 211 259 268 8585957 10.1007/978-3-642-85232-9_26

[B126] Zeleznik-Le NJ Harden AM Rowley JD 11q23 translocations split the "AT-hook" cruciform DNA-binding region and the transcriptional repression domain from the activation domain of the mixed-lineage leukemia (MLL) gene Proc Natl Acad Sci USA 1994 91 22 10610 10614 10.1073/pnas.91.22.10610 7938000 PMC45071

[B127] Ozgenc A Loeb LA Current advances in unraveling the function of the Werner syndrome protein Mutat Res 2005 577 1-2 237 251 15946710 10.1016/j.mrfmmm.2005.03.020

[B128] Hanada K Hickson ID Molecular genetics of RecQ helicase disorders Cell Mol Life Sci 2007 64 17 2306 2322 10.1007/s00018-007-7121-z 17571213 PMC11136437

[B129] Compton SA Tolun G Kamath-Loeb AS Loeb LA Griffith JD The Werner syndrome protein binds replication fork and holliday junction DNAs as an oligomer J Biol Chem 2008 283 36 24478 24483 10.1074/jbc.M803370200 18596042 PMC2528990

[B130] Chan TA Hermeking H Lengauer C Kinzler KW Vogelstein B 14-3-3Sigma is required to prevent mitotic catastrophe after DNA damage Nature 1999 401 6753 616 620 10.1038/44188 10524633

[B131] Chu WK Hickson ID RecQ helicases: multifunctional genome caretakers Nat Rev Cancer 2009 9 9 644 654 10.1038/nrc2682 19657341

[B132] Jett SD Cherny DI Subramaniam V Jovin TM Scanning force microscopy of the complexes of p53 core domain with supercoiled DNA J Mol Biol 2000 299 3 585 592 10.1006/jmbi.2000.3759 10835269

[B133] Iwasaki H Takahagi M Shiba T Nakata A Shinagawa H Escherichia coli RuvC protein is an endonuclease that resolves the Holliday structure Embo J 1991 10 13 4381 4389 1661673 10.1002/j.1460-2075.1991.tb05016.x PMC453191

[B134] Biertumpfel C Yang W Suck D Crystal structure of T4 endonuclease VII resolving a Holliday junction Nature 2007 449 7162 616 620 10.1038/nature06152 17873859

[B135] Pan PS Curtis FA Carroll CL Medina I Liotta LA Sharples GJ McAlpine SR Novel antibiotics: C-2 symmetrical macrocycles inhibiting Holliday junction DNA binding by E. coli RuvC Bioorg Med Chem 2006 14 14 4731 4739 10.1016/j.bmc.2006.03.028 16581254

[B136] Fogg JM Schofield MJ Declais AC Lilley DM Yeast resolving enzyme CCE1 makes sequential cleavages in DNA junctions within the lifetime of the complex Biochemistry 2000 39 14 4082 4089 10.1021/bi992785v 10747798

[B137] Garcia AD Otero J Lebowitz J Schuck P Moss B Quaternary structure and cleavage specificity of a poxvirus holliday junction resolvase J Biol Chem 2006 281 17 11618 11626 10.1074/jbc.M600182200 16513635

[B138] Biswas T Aihara H Radman-Livaja M Filman D Landy A Ellenberger T A structural basis for allosteric control of DNA recombination by lambda integrase Nature 2005 435 7045 1059 1066 10.1038/nature03657 15973401 PMC1809751

[B139] Declais AC Liu J Freeman AD Lilley DM Structural recognition between a four-way DNA junction and a resolving enzyme J Mol Biol 2006 359 5 1261 1276 10.1016/j.jmb.2006.04.037 16690083

[B140] Guan C Kumar S A single catalytic domain of the junction-resolving enzyme T7 endonuclease I is a non-specific nicking endonuclease Nucleic Acids Res 2005 33 19 6225 6234 10.1093/nar/gki921 16264086 PMC1277808

[B141] Hadden JM Declais AC Carr SB Lilley DM Phillips SE The structural basis of Holliday junction resolution by T7 endonuclease I Nature 2007 449 7162 621 624 10.1038/nature06158 17873858

[B142] Spiro C McMurray CT Switching of DNA secondary structure in proenkephalin transcriptional regulation J Biol Chem 1997 272 52 33145 33152 10.1074/jbc.272.52.33145 9407101

[B143] Middleton CL Parker JL Richard DJ White MF Bond CS Substrate recognition and catalysis by the Holliday junction resolving enzyme Hje Nucleic Acids Res 2004 32 18 5442 5451 10.1093/nar/gkh869 15479781 PMC524281

[B144] Lyu YL Lin CT Liu LF Inversion/dimerization of plasmids mediated by inverted repeats J Mol Biol 1999 285 4 1485 1501 10.1006/jmbi.1998.2419 9917391

[B145] Giraud-Panis MJ Lilley DM Near-simultaneous DNA cleavage by the subunits of the junction-resolving enzyme T4 endonuclease VII Embo J 1997 16 9 2528 2534 10.1093/emboj/16.9.2528 9171365 PMC1169852

[B146] Macmaster R Sedelnikova S Baker PJ Bolt EL Lloyd RG Rafferty JB RusA Holliday junction resolvase: DNA complex structure--insights into selectivity and specificity Nucleic Acids Res 2006 34 19 5577 5584 10.1093/nar/gkl447 17028102 PMC1636454

[B147] Owen BA W HL McMurray CT The nucleotide binding dynamics of human MSH2-MSH3 are lesion dependent Nat Struct Mol Biol 2009 16 5 550 557 10.1038/nsmb.1596 19377479 PMC2982795

[B148] Surtees JA Alani E Mismatch repair factor MSH2-MSH3 binds and alters the conformation of branched DNA structures predicted to form during genetic recombination J Mol Biol 2006 360 3 523 536 10.1016/j.jmb.2006.05.032 16781730

[B149] Chang JH Kim JJ Choi JM Lee JH Cho Y Crystal structure of the Mus81-Eme1 complex Genes Dev 2008 22 8 1093 1106 10.1101/gad.1618708 18413719 PMC2335329

[B150] Ehmsen KT Heyer WD Saccharomyces cerevisiae Mus81-Mms4 is a catalytic, DNA structure-selective endonuclease Nucleic Acids Res 2008 36 7 2182 2195 10.1093/nar/gkm1152 18281703 PMC2367710

[B151] Taylor ER McGowan CH Cleavage mechanism of human Mus81-Eme1 acting on Holliday-junction structures Proc Natl Acad Sci USA 2008 105 10 3757 3762 10.1073/pnas.0710291105 18310322 PMC2268786

[B152] Fouche N Cesare AJ Willcox S Ozgur S Compton SA Griffith JD The basic domain of TRF2 directs binding to DNA junctions irrespective of the presence of TTAGGG repeats J Biol Chem 2006 281 49 37486 37495 10.1074/jbc.M608778200 17052985

[B153] Lee JH Park CJ Arunkumar AI Chazin WJ Choi BS NMR study on the interaction between RPA and DNA decamer containing cis-syn cyclobutane pyrimidine dimer in the presence of XPA: implication for damage verification and strand-specific dual incision in nucleotide excision repair Nucleic Acids Res 2003 31 16 4747 4754 10.1093/nar/gkg683 12907715 PMC169961

[B154] Sekelsky JJ Hollis KJ Eimerl AI Burtis KC Hawley RS Nucleotide excision repair endonuclease genes in Drosophila melanogaster Mutat Res 2000 459 3 219 228 10812334 10.1016/s0921-8777(99)00075-0

[B155] Lee S Cavallo L Griffith J Human p53 binds Holliday junctions strongly and facilitates their cleavage J Biol Chem 1997 272 11 7532 7539 10.1074/jbc.272.11.7532 9054458

[B156] Ma B Levine AJ Probing potential binding modes of the p53 tetramer to DNA based on the symmetries encoded in p53 response elements Nucleic Acids Res 2007 35 22 7733 7747 10.1093/nar/gkm890 17986463 PMC2190717

[B157] Mullen JR Nallaseth FS Lan YQ Slagle CE Brill SJ Yeast Rmi1/Nce4 controls genome stability as a subunit of the Sgs1-Top3 complex Mol Cell Biol 2005 25 11 4476 4487 10.1128/MCB.25.11.4476-4487.2005 15899853 PMC1140617

[B158] Rass U Kemper B Crp1p, a new cruciform DNA-binding protein in the yeast Saccharomyces cerevisiae J Mol Biol 2002 323 4 685 700 10.1016/S0022-2836(02)00993-2 12419258

[B159] van Houte LP Chuprina VP van der Wetering M Boelens R Kaptein R Clevers H Solution structure of the sequence-specific HMG box of the lymphocyte transcriptional activator Sox-4 J Biol Chem 1995 270 51 30516 30524 10.1074/jbc.270.51.30516 8530483

[B160] Pearson CE Ruiz MT Price GB Zannis-Hadjopoulos M Cruciform DNA binding protein in HeLa cell extracts Biochemistry 1994 33 47 14185 14196 10.1021/bi00251a030 7947830

[B161] Nakamura Y Yoshioka K Shirakawa H Yoshida M HMG box A in HMG2 protein functions as a mediator of DNA structural alteration together with box B J Biochem 2001 129 4 643 651 11275566 10.1093/oxfordjournals.jbchem.a002902

[B162] Culard F Gervais A de Vuyst G Spotheim-Maurizot M Charlier M Response of a DNA-binding protein to radiation-induced oxidative stress J Mol Biol 2003 328 5 1185 1195 10.1016/S0022-2836(03)00361-9 12729751

[B163] Tripathi P Pal D Muniyappa K Saccharomyces cerevisiae Hop1 protein zinc finger motif binds to the Holliday junction and distorts the DNA structure: implications for holliday junction migration Biochemistry 2007 46 44 12530 12542 10.1021/bi701078v 17935355

[B164] Tripathi P Anuradha S Ghosal G Muniyappa K Selective binding of meiosis-specific yeast Hop1 protein to the holliday junctions distorts the DNA structure and its implications for junction migration and resolution J Mol Biol 2006 364 4 599 611 10.1016/j.jmb.2006.08.096 17027027

[B165] Rene B Fermandjian S Mauffret O Does topoisomerase II specifically recognize and cleave hairpins, cruciforms and crossovers of DNA? Biochimie 2007 89 4 508 515 10.1016/j.biochi.2007.02.011 17397986

[B166] Dip R Naegeli H More than just strand breaks: the recognition of structural DNA discontinuities by DNA-dependent protein kinase catalytic subunit Faseb J 2005 19 7 704 715 10.1096/fj.04-3041rev 15857885

[B167] Bonnefoy E Takahashi M Yaniv JR DNA-binding parameters of the HU protein of Escherichia coli to cruciform DNA J Mol Biol 1994 242 2 116 129 10.1006/jmbi.1994.1563 8089835

[B168] Pinson V Takahashi M Rouviere-Yaniv J Differential binding of the Escherichia coli HU, homodimeric forms and heterodimeric form to linear, gapped and cruciform DNA J Mol Biol 1999 287 3 485 497 10.1006/jmbi.1999.2631 10092454

